# Supplementary material for: APOE Locus-Associated Mitochondrial Function and Its Implication in Alzheimer’s Disease and Aging
Source: Int J Mol Sci. 2023 Jun 21;24(13):10440. doi: 10.3390/ijms241310440 (PMC10341489; doi:10.3390/ijms241310440)

***APOE* Locus-Associated Mitochondrial Function and Its Implication in Alzheimer's Disease and Aging**

**Supplementary Tables and Figures**

**Table S1.** Primers, probes, and TaqMan assays.

**Figure S1.** Alterations of apoptosis-related gene expression during the $H_2O_2$ recovery phase.

**Figure S2.** Variations of mitochondrial structure and function-related gene expression in human PMB tissues.

**Figure S3.** Variations of mitochondrial DNA (mtDNA) copy numbers in human PMB tissues.

**Figure S4.** Three-dimensional (3D) genome structure of the *APOE* locus.

**Table S1.** Primers, probes, and TaqMan assays.

| Method | Target | Primer | Sequence 5'- | Amplicon (bp) | Comment |
|---|---|---|---|---|---|
| **SYBR-based PCR** | | | | | |
| | *MT-ND1* | MT-ND1_F | CCTACTCCTCATTGTACCCATTC | 93 | Forward |
| | | MT-ND1_R | GGGCCTTTGCGTAGTTGTA | | Reverse |
| | *NCOA3* | NCOA3_F | GCCATACATTTAATTGCCGTATGT | 94 | Forward |
| | | NCOA3_R | GTTTCATATCTCTGGCGCATTTC | | Reverse |
| | *REST* | REST_F | GCGTACTCATTCAGGTGAGAAG | 113 | Forward |
| | | REST_R | GAGGTTTAGGCCCATTGTGAA | | Reverse |
| | *SOD1* | SOD1_F | GTGCAGGGCATCATCAATTTC | 85 | Forward |
| | | SOD1_R | GGCCTTCAGTCAGTCCTTTAAT | | Reverse |
| | *SIRT1* | SIRT1_F | GTAGGCGGCTTGATGGTAAT | 101 | Forward |
| | | SIRT1_R | GGATAAGACGTCATCTTCAGAGTC | | Reverse |
| | *CASP3* | CASP3_F | ATGGATTATCCTGAGATGGGTTTAT | 89 | Forward |
| | | CASP3_R | TCTGTACCAGACCGAGATGT | | Reverse |
| | *CRYAB* | CRYAB_F | TGGGAGATGTGATTGAGGTG | 99 | Forward |
| | | CRYAB_R | CAGCTGGGATCCGGTATTT | | Reverse |
| | *MFN1* | MFN1_F | AACCTGGCAGCTGAAGATAAA | 83 | Forward |
| | | MFN1_R | TGGTTTCGAATAAAGTCCAGTCT | | Reverse |
| | *FIS1* | FIS1_F | TGCGGAGCAAGTACAATGATG | 97 | Forward |
| | | FIS1_R | AAGACGTAATCCCGCTGTTC | | Reverse |
| | *DNM1L* | DNM1L_F | AGCTCCAGGACGTCTTCAAC | 79 | Forward |
| | | DNM1L_R | CTGCTCTGCGTTCCCACTAC | | Reverse |
| | *PINK1* | PINK1_F | TCGGCCTGTCAGGAGATC | 78 | Forward |
| | | PINK1_R | CAAGCGTCTCGTGTCCAAC | | Reverse |
| | *TFAM* | TFAM_F | AAAGACCTCGTTCAGCTTATAAC | 94 | Forward |
| | | TFAM_R | TCCTTTACAGTCTTCAGCTTTTC | | Reverse |
| | *ACTB* | ACTB_F | GGAATCCTTCTGACCCATGCC | 70 | Forward |
| | | ACTB_R | CGTCTTCCCCTCCATCGTGG | | Reverse |
| **Digital PCR** | | | | | |
| | *TOMM40* | TOMM40_F2 | TCAGCACAATCGGGGAGTC | 112 | Forward |
| | | TOMM40_R2 | CCACTGTTGTCCATGTCACCC | | Reverse |
| | | TOMM40 probe | ACAAAGCAGCTGAGTCCCACAGAG | | Internal probe |
| | *ACTB* | ACTB_F | TCGCCCACATAGGAATCCTTC | 81 | Forward |
| | | ACTB_R | CGTCTTCCCCTCCATCGTGG | | Reverse |
| | | ACTB probe | TGACCCATGCCCACCATCAC | | Internal probe |
| ***TOMM40* Allelic Expression** | | | | | |
| | *TOMM40* | TOMM40_F1-Bio | Bio-AGATGGAGGGTGTCAAG | 141 | Forward |
| | | TOMM40_R1 | TGGGACTCAGCTGCTTTG | | Reverse |
| **TaqMan Assay** | | | | | |
| | *APOE* RNA | | ThermoFisher (Hs00171168_m1) | | |
| | *APOC1* RNA | | ThermoFisher (Hs00155790_m1) | | |
| | *NECTIN2* RNA | | ThermoFisher (Hs01071562_m1) | | |
| | *TOMM40* RNA | | ThermoFisher (Hs01587378_mH) | | |
| | *ACTB* RNA | | ThermoFisher (Hs01060665_g1) | | |

**Supplementary Figure S1.** Alterations of apoptosis-related gene expression during the $H_2O_2$ recovery phase. RNA levels of two genes, *CASP3* (A) and *CRYAB* (B) were quantified in experiment condition cells by RT-qPCR (SYBR-based). Fold changes (FC) to their untreated counterparts (set as baseline of 1.0) are plotted as average and standard deviation of three to four independent experiments. The first culture condition, c1, represents cells treated with $H_2O_2$ for 24 h; c2, cells in $H_2O_2$ recovery phase after replenishing culture with fresh media and continuing to culture for additional 24 h; c3, cells in $H_2O_2$ recovery phase for culturing additional 48 h.

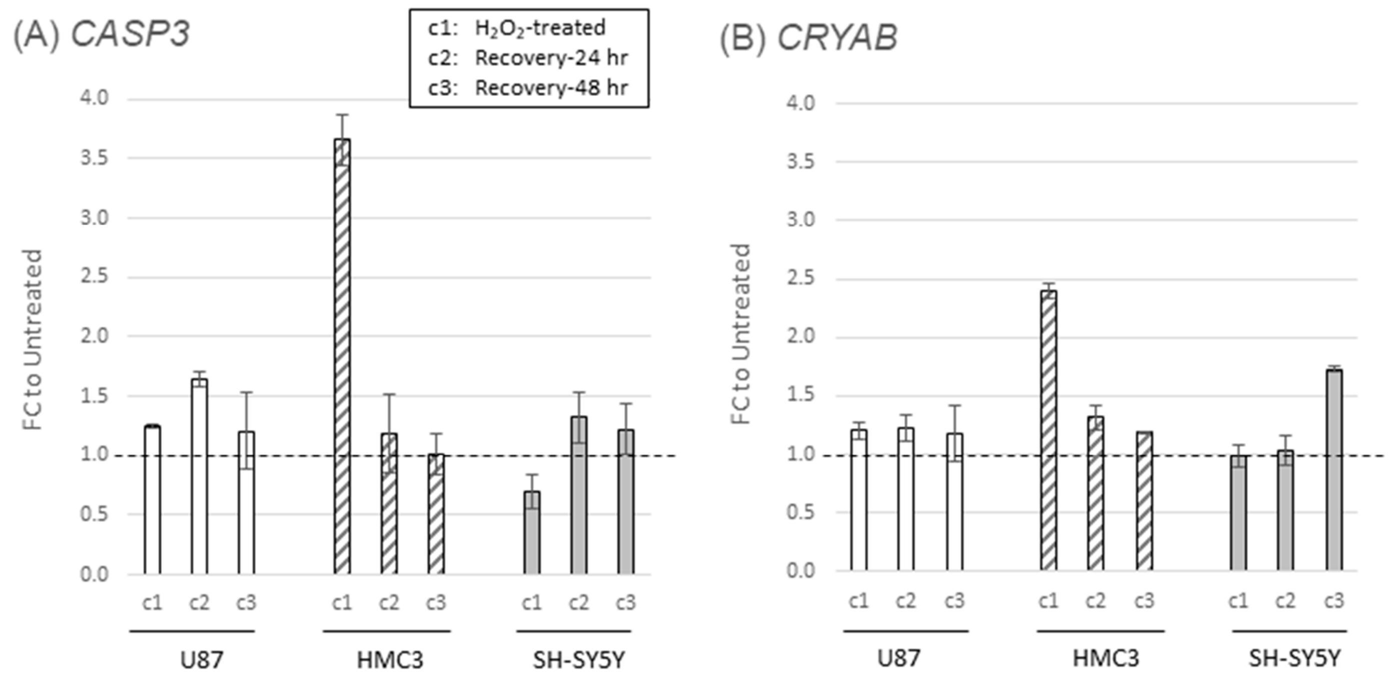

**Supplementary Figure S2.** Variations of mitochondrial structure and function-related gene expression in human PMB tissues. Using a subgroup of PMB samples [AD (n=14) and control (CTRL, n=10)] RNA levels were quantified by RT-qPCR (SYBR-based). (A) Antioxidant response genes, (B) Apoptosis genes, (C) Mitochondrial dynamics genes, and (D) a mtDNA maintenance gene. The ΔCt method was used, in which a larger ΔCt value indicates a higher RNA level. RNA levels of AD (grey filled) were compared with those of control (unfilled) by independent samples t-test using R-Program; *, $p<0.05$.

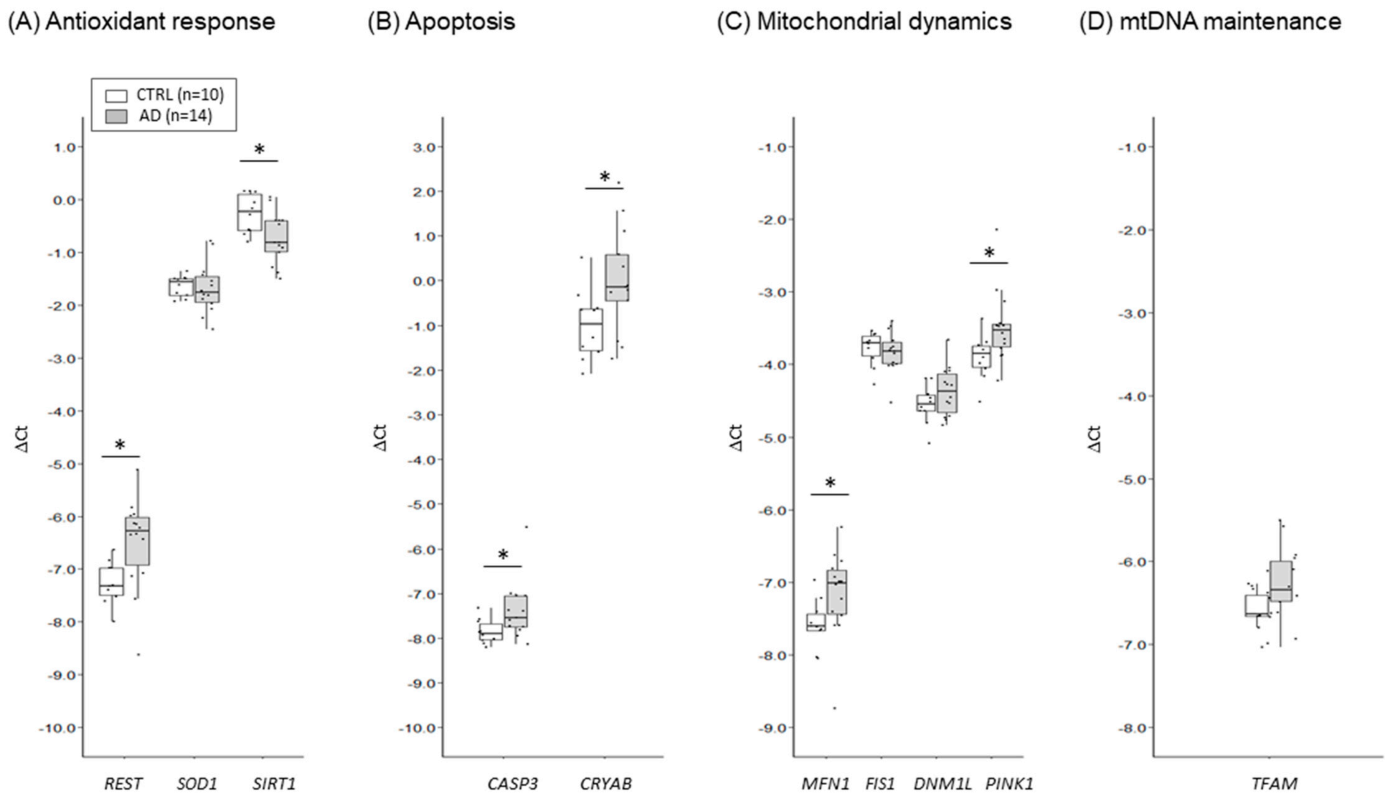

**Supplementary Figure S3.** Variations of mitochondrial DNA (mtDNA) copy numbers in human PMB tissues. Using PMB AD and control (CTRL) samples mtDNA copy numbers were quantified by RT-qPCR (SYBR-based). mtDNA copy numbers are stratified by alleles of SNPs: *APOE* rs429358 (A), *APOC1* rs11568822 (B) and *TOMM40* rs2075650 (C). The normalized ΔCt shows that a larger ΔCt value indicates a greater number of mtDNA. Independent samples t-test p-values are indicated. Numbers in parentheses denote sample sizes.

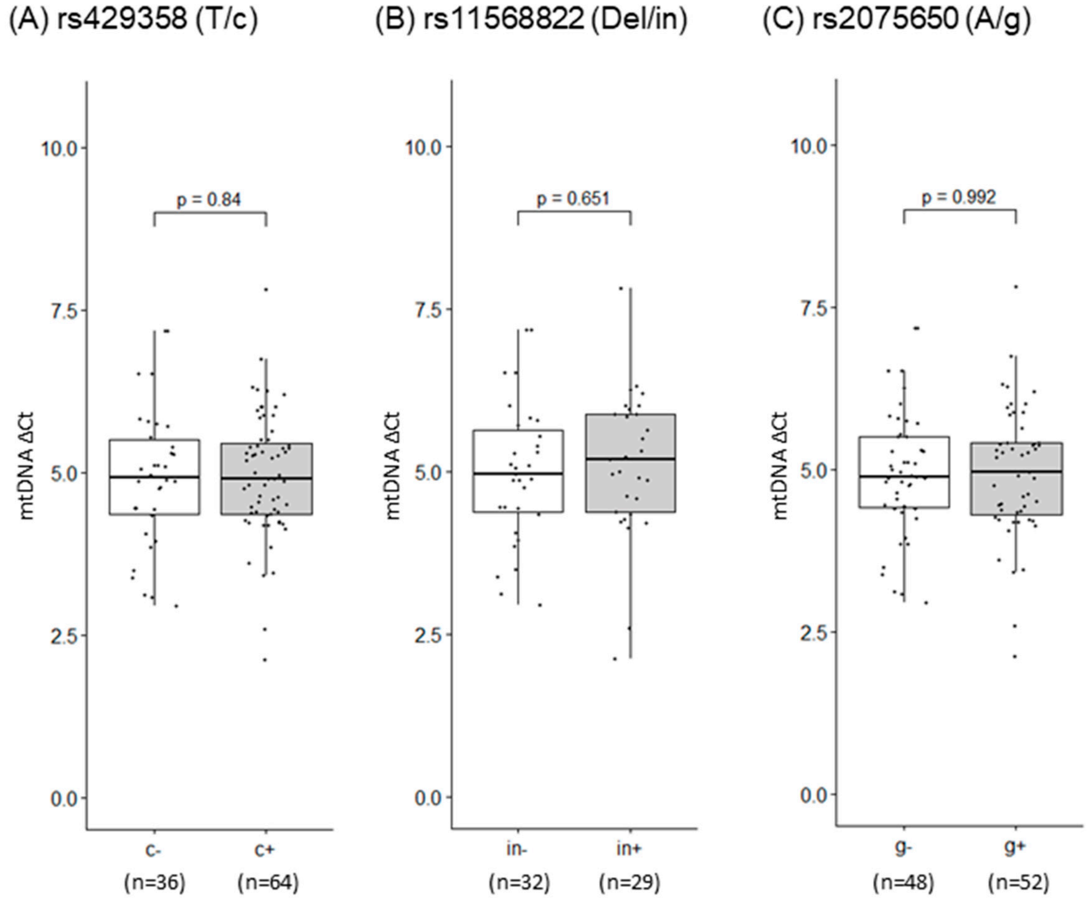

**Supplementary Figure S4.** Three-dimensional (3D) genome structure of the *APOE* locus. The UCSC Genome Browser's Hi-C/Micro-C tracks of the *APOE* locus. (A) a display of the entire region of *APOE* locus with a black triangle that is (B) a zoomed-in core region with blue triangles showing regions of strong interaction. This track provides heatmaps of chromatin folding data from in situ Hi-C and Micro-C XL experiments on the H1-hESC (embryonic stem cells) and HFFc6 (foreskin fibroblasts) cell lines. The data indicate how many interactions were detected between regions of the genome. A high score between two regions suggests that they are in close proximity in 3D space within the nucleus, which is shown by a more intense color in the heatmap. The color shade at any point within the triangle shows the proximity score for two genomic regions.

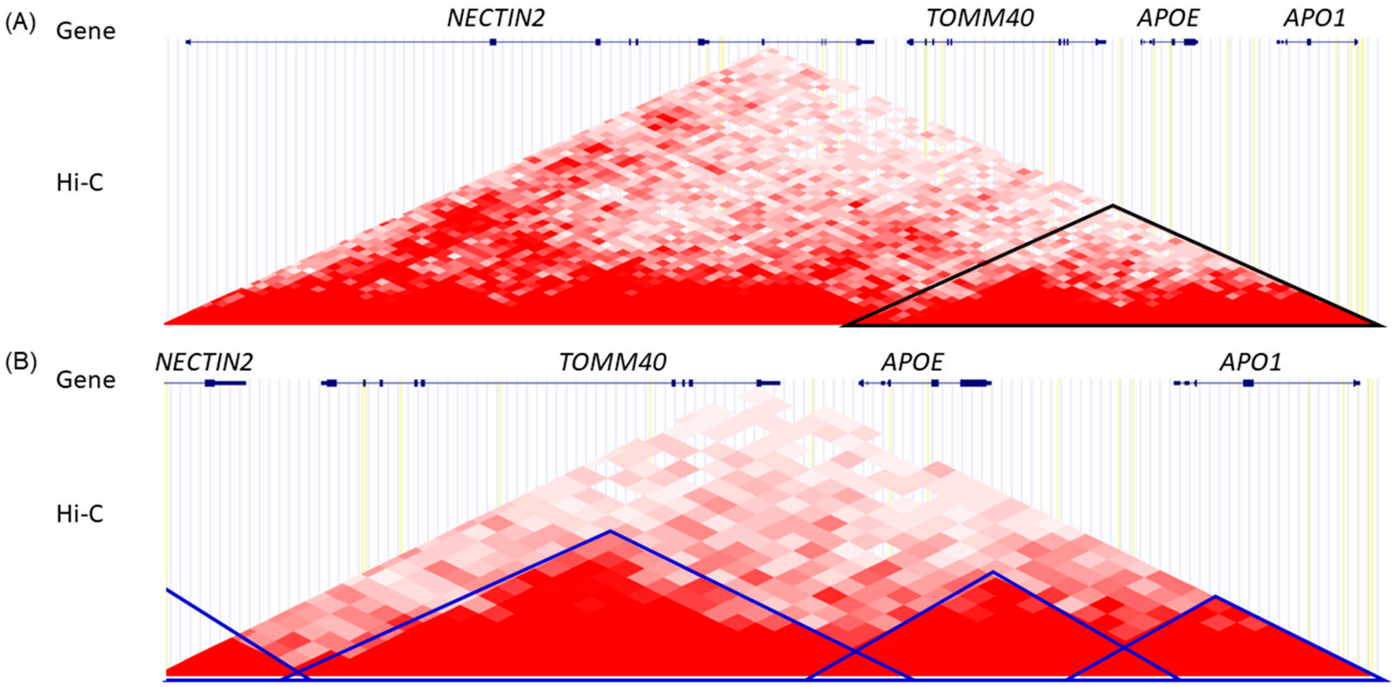

Supplement: Supplementary file 1 [file ijms-24-10440-s001.zip › ijms-2397016-supplementary.pdf]
